# Dual-Layer Rotation: A Versatile Therapeutic Mammoplasty Technique

**DOI:** 10.1245/s10434-022-11977-4

**Published:** 2022-06-16

**Authors:** Sarianna Joukainen, Elina Laaksonen, Ritva Vanninen, Outi Kaarela, Mazen Sudah

**Affiliations:** 1 Division of Plastic Surgery, Department of Surgery, Kuopio University Hospital, Kuopio, Finland; 2 Diagnostic Imaging Center, Department of Clinical Radiology, Kuopio University Hospital, Kuopio, Finland; 3 Cancer Center of Eastern Finland, University of Eastern Finland, Kuopio, Finland

## Abstract

**Background:**

Multifocal or complex breast lesions are a challenge for breast-conserving surgery, particularly surgery in small breasts or those located in the upper inner quadrant. The dual-layer rotation technique exploits the idea of manipulating the skin and glandular tissue in separate layers to fill the resection cavity via vertical mammoplasty if skin excision is not required, except in the central area.

**Methods:**

The authors performed a retrospective review of consecutive breast cancer patients who underwent DLR mammoplasty between 2017 and 2019 at a single institution. Clinical data, reoperations, surgical complications, delays in adjuvant treatments, and the need for late revisional surgery were evaluated. Aesthetic outcomes were evaluated objectively and subjectively from photographs.

**Results:**

The study included 46 breasts of 40 patients. Tumors were located in the UIQ (30%, 14/46) or in multiple quadrants (22%, 10/46). One third (33%, 13/40) of the patients had a small breast cup size (A–B). Negative margins were primarily achieved in 45 of the 46 breasts. Major complications occurred in three patients, who needed reoperation, and adjuvant therapy was delayed for one of these patients. Late refinement surgery was needed for two patients. The objective and subjective aesthetic outcomes were good or excellent regardless of the tumor position.

**Conclusion:**

As a novel oncoplastic approach, DLR mammoplasty offers a one-step procedure to treat selected breast cancer patients with challenging resection defects due to different breast sizes or lesion locations. The technique preserves the breast’s natural appearance.

Breast-conserving surgery (BCS) is the gold standard of care in the management of most patients with early breast cancer.^1,2^ Oncoplastic breast-conserving surgery (OBCS), in which plastic surgery techniques are applied to breast cancer surgery, have broadened the possibilities of conserving the breast without compromising aesthetic results, even for patients with large tumors, multifocal lesions, or lesions in unfavorable locations such as the upper inner quadrant (UIQ) or central areas.^3^ Furthermore, OBCS was shown to be a safe oncologic procedure and provided outcomes comparable with those of standard BCS but offered improved aesthetic and quality-of-life outcomes as well as a reduction in the reoperation rate due to insufficient margins.^4^ The medial half of the breast is less tolerant to volume loss, and removing more than 8% to 10% of the tissue volume might necessitate oncoplastic techniques to avoid suboptimal cosmetic outcomes.^5^ Lesions located in the UIQ particularly represent a cosmetic challenge for OBCS, especially those in smaller breasts or breasts with multifocal or complex lesions.^6,7^

In OBCS, the tumor bed resection cavity can be corrected by displacement techniques using the remaining breast tissue. This usually is applied in large or ptotic breasts or can be replaced with flaps if the remaining breast volume is not sufficient for displacement. Numerous displacement techniques have been published. They often use mammoplasty or mastopexy, parenchymal remodeling, or local tissue rearrangement techniques to fill the tumor cavity.^3,8^ Parenchymal rotation can be achieved under the intact overlying skin if the defect is small.^9^ Parenchymal rotation also can be combined with the skin,^10,11^ permitting larger resections in peripheral locations, but may result in extensive visible scars.

Dual layer rotation therapeutic mammoplasty is a one-step procedure for treating selected breast cancer patients with challenging resection defects in various locations, including the upper inner quadrant and in smaller breasts, and helps to preserve a natural aesthetic appearance.

Vertical opening-based oncoplastic mammoplasty can reduce the scar burden and optimize the location of the scar. Tissues that typically are removed during mammaplasty techniques can be repositioned to compensate for the volume loss at the adjacent site of the excised tumor area. Several oncoplastic procedures advance and rotate excess skin and glandular tissues at the 6 o’clock position during vertical mammoplasty to the central or medio-cranial position^12,13^ and transpose those to the lateral tumor bed.^14^

The breast has multiple sources of blood supply. The main inflows include the internal mammary segmental perforators, the anteromedial and lateral intercostal perforators, and the external mammary artery. Breast remodeling of different layers including the skin envelope, breast parenchyma, and nipple areola complex (NAC) as separate or combined units is possible providing the vascularity to each region is preserved. Greater parenchymal rotation without the skin may be possible, mimicking Corduff’s mastopexy technique,^15^ not only in a lateral direction but also to all the inferior, central, and medial parts, assuming the lateral vessels are preserved.

The current study investigated a novel application of an oncoplastic technique, dual-layer rotation (DLR), which combines the principles of both rotation mammoplasty and vertical mammoplasty. The parenchyma is rotated to fill the tumor defect in one layer, and the overlying skin is rotated in the opposite direction in another layer from the 6 o’clock position to compensate for the geometric volume. We evaluated the clinical and aesthetic outcomes of breast cancer patients who underwent DLR OBCS with variable, predominantly unfavorable tumor locations, or for multifocal lesions requiring large resection.

## Materials and Methods

### Ethics

The Chair of the hospital district waived the need to obtain written informed consent from the patients due to the retrospective nature of the analyses. All clinical investigations were conducted according to the relevant guidelines and the principles of the Declaration of Helsinki.

### Study Population

**Fig. 1:**
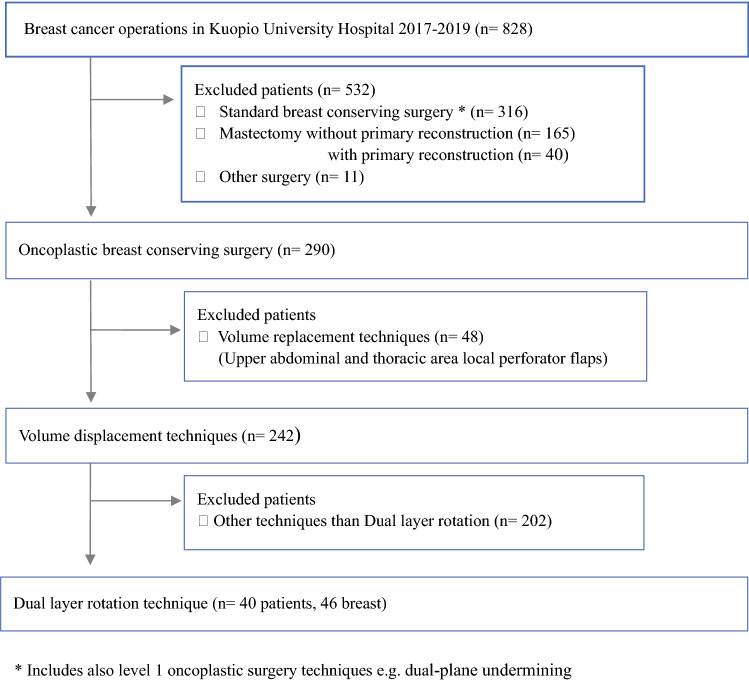
Flow chart showing study population of dual-layer rotation technique

The study population comprised all consecutive breast cancer patients who underwent DLR mammoplasty out of 828 new breast cancer cases in Kuopio University Hospital between January 2017 and December 2019 (Fig. 1). The DLR technique was used for the patients who did not require skin excision above the tumor unless skin removal was in the novel areola placement. All DLR patients’ excisional and reconstructive procedures were performed by three separate plastic surgeons.

The patient, tumor, surgical, and adjuvant treatment characteristics were retrieved from the local electronic medical records. Reoperations, surgical complications, delays in adjuvant treatments, the need for late revisional surgery, and the aesthetic result were also recorded.

The Clavien–Dindo classification (CDC) adapted for breast cancer^16^ was used to classify surgical complications, and they were divided into minor and major types. Major complications were defined as those that required hospital readmission or a return to the operating room. Adjuvant treatment was considered delayed if it was started more than 8 weeks after primary surgery. Fat necrosis was defined as a palpable, discrete, and persistent subcutaneous firmness found postoperatively measuring at least 1 cm during physical examination^17^ and confirmed radiologically.

Cosmetic results were evaluated using photographs taken 1 to 2 years after oncologic treatments by two methods: objectively using Breast Cancer Conservation Treatment.cosmetic results (BCCT.core) software^18^ and subjectively by a nonpartisan surgeon who used Harvard scale.^19^ Using both methods, the scales were converted to numbers: 4 (excellent), 3 (good), 2 (fair), and 1 (poor).

### Surgery

#### Preoperative Planning

The extent of the breast tissue resection area was planned for individual patients in multidisciplinary meetings taking into consideration the clinical, radiologic, and histologic findings, as well as the patient’s preference. Guidewire localization of nonpalpable lesions was performed under ultrasound or stereotactic guidance by a specialized breast radiologist, who ink-marked the skin to show the location and extent of the lesions to be resected in the surgical position, including supine magnetic resonance imaging (MRI)-guided localization projections.^20^

**Fig. 2:**
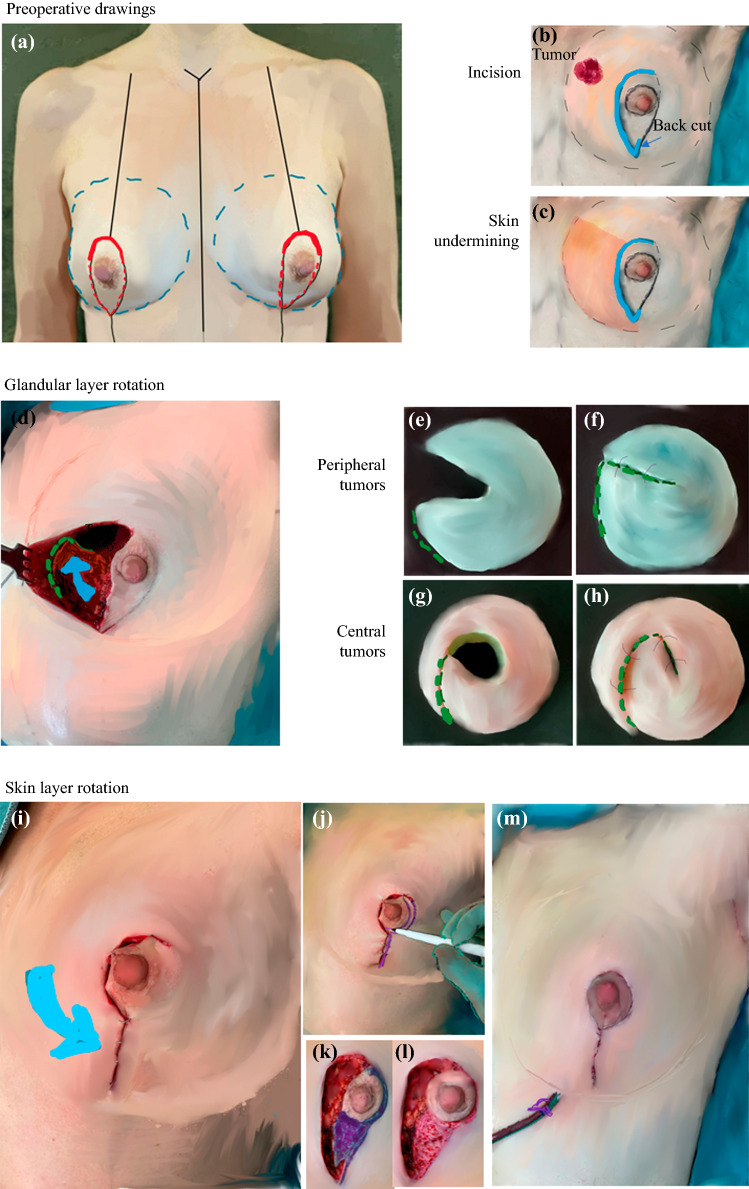
Illustration of the dual-layer rotation mammoplasty technique. **a** Preoperative drawings with the subject in the upright position. The footprint is shown with a dashed blue line. The meridian of the breast and mid-torso is shown in black, the desired highest part of the areola in red, and the vertical opening sketch in a dashed red line. **b** The skin incision is made to the vertical limb only on the tumor side, and a 1-cm back cut is made to the bottom of the opposite vertical limb. **c** The skin is released from the parenchyma above the tumor down to the vertical opening. **d** After removal of the tumor, the glandular layer is excised from the peripheral end of the tumor and curved toward the vertical opening until the flap can be rotated (*blue arrow*) to fill the tumor defect. **e**–**h** Schematic drawings showing how the glandular tissue is rotated in patients with peripheral tumors (**e**, **f**) or central tumors (**g**, **h**). **i**, **j** The skin layer is rotated (**i:** *blue arrow*) in the opposite direction toward the meridian of the breast and temporarily fixed (**j**). **k** Excess skin is ink-marked in purple. **l**, **m** Illustration of the ink-marked area after release of the temporary fixation and de-epithelization (**l**) followed by skin closure and drainage (**m**)

The orientation drawings for OBCS were marked preoperatively by surgeons with the patient in the upright position. The midline of the torso, footprint, and ideal breast meridian were marked. The place of the desired NAC was evaluated in proportion to the chest height and width, and the anticipated upper line of the areola was marked. From this mark, a downward narrowing ellipse was drawn to the inframammary crease or 1 to 2 cm above it for large or ptotic breasts requiring high-volume resection (Fig. 2a).

#### Tumor Resection

A half elliptical vertical skin incision was made on the tumor side, and an approximately 1-cm back-cut was made to the caudal part of the contralateral vertical limb. The skin was freed from the underlying glandular tissue from the tumor resection area down to the inframammary crease (Fig. 2b). Undermining of the skin over the tumor was performed, as in mastectomy, leaving a skin flap with a maximum thickness of 5 mm (Fig. 2c). Full-thickness fibroglandular tissue resection was performed from the subcutaneous area to the muscle, including the pectoral fascia. Tumors were excised en bloc with a healthy macroscopic surgical margin of at least 1 cm in accordance with national guidelines, thereby achieving microscopically negative margins, defined as no “tumor-on-ink” for patients with invasive cancers and a 1- to 2-mm margin for patients with ductal carcinoma *in situ* (DCIS). The skin was excised only if deemed necessary and if it was situated in the novel NAC area. The tumor bed was marked with clips. Breast tissue specimens were marked with orientation clips, weighed, and fixed to styrofoam slabs in the anatomic position. Specimen radiographs and ultrasound also were performed if intraoperative radiologic confirmation of the adequacy of tumor resection and margins was required

#### Axillary Procedures

Sentinel lymph node biopsy was performed as a staging procedure for patients with invasive carcinoma or large DCIS. Axillary lymph node dissection was performed if axillary metastasis was histologically verified preoperatively or diagnosed intraoperatively using frozen sections. Where necessary, frozen sections were obtained for the most active nodes and for clinically suspicious lymph nodes in accordance with national guidelines.

#### Reshaping the Breast With the DLR Technique

##### Glandular Layer (Fig. 2D–H)

Glandular tissue was split as needed until the tissue rotation filled the resection cavity. The spilt line started from the peripheral end of the resection cavity and curved toward the lowest point of the skin opening in the inframammary crease. Subglandular undermining under the rotational flap was performed only if needed. For patients with peripheral tumor, glandular rotation was performed directly, whereas a spiral shape was used to fill the resection cavity in patients with more central defects. The glandular flap was fixed to the surrounding breast tissue with multifilament absorbable sutures.

##### Skin Layer (Fig. 2I–M)

To adjust the skin envelope to the breast, the previously released skin was rotated to the breast meridian and fixed temporarily with a few staples. The excess skin in the vertical opening and areola area was marked and de-epithelialized after removal of the staples. Skin was preserved in the central area to form a neo-areola in patients with retro-areolar tumors who required NAC removal. The skin was gathered using interrupted 3.0 or 4.0 absorbable sutures, and skin closure was completed with 4.0 monofilament barbed or non-barbed continuous sutures. Drains were used until fluid formation was less than 40 ml/day.

### Nipple Reconstruction

If the nipple was to be removed, reconstruction was offered and performed either immediately or at a later time depending on the patient’s preference. The technique used depended on the position of the excess skin. Tattooing was performed 6 months after adjuvant radiotherapy.

### Healthy Breast

Patients were offered mastopexy or mammoplasty to match the healthy breast if obvious asymmetry was expected, which was performed for those patients who elected to undergo contralateral breast symmetrization procedures. Vertical opening was used, and NAC vascularity was preserved in the superomedial direction

### Histopathology

Pathologists received an orientation resection map, which was first drawn by radiologists and complemented by the surgeon. The surfaces of breast specimens were color-inked for orientation, dissected at 5-mm intervals, and fixed in formalin. The histopathologic dimensions, type, grade, and margins of the tumor were evaluated by experienced breast pathologists based on the World Health Organization classification of tumors.^21^ Hormone receptor status and the Ki-67 proliferation index were assessed by immunohistochemistry for all invasive lesions, and the human epidermal growth factor receptor-2 (HER2) oncogene amplification status was confirmed by chromogen *in situ* hybridization. Formalin-fixed lymph nodes were serially sectioned into 2-mm-thick slices, which were embedded in paraffin and stained with hematoxylin and eosin. If metastasis was not detected, immunohistochemical staining was performed against low-molecular-weight cytokeratin to detect micrometastasis.

## Results

### Patients

The DLR technique was performed for 40 patients (all Caucasian) and 46 breasts. Overall, 32 patients underwent bilateral breast surgery due to either synchronous DCIS or invasive carcinoma (*n* = 4), risk or benign lesions (*n* = 2), or for symmetrization (*n* = 26). Eight patients did not wish to undergo contralateral symmetrization. The patient and tumor characteristics as well as the surgical procedures are summarized in Table 1.

**Table 1:** Patient, tumor, and treatment characteristics

Patient, tumor, and treatment characteristics		Patients (*n* = 40) *n* (%)	Breasts (*n* = 46) *n* (%)
Patient	Mean age: years (range)	58.6 (33–79)	
	Mean BMI: kg/m^2^ (range)	25.5 (19.4–35.6)	
	Smoking history		
	Nonsmoker	28 (70)	
	Ex-smoker	10 (25)	
	Current smoker	2 (5)	
	Cup size		
	A–B	13 (33)	
	C–D	17 (43)	
	E/larger	6 (15)	
	Missing	4 (10)	
Surgery	Bilateral breast surgery		
	Total	32 (80)	
	Reason for bilateral surgery		
	Bilateral carcinoma or DCIS	4 (10)	
	Risk or benign lesion	2 (5)	
	Symmetrization	26 (65)	
	No symmetrization	8 (20)	
	Lymph node surgery		
	No surgery		8 (17)
	SLNB		32 (70)
	SNLB and axillary clearance		3 (7)
	Axillary clearance		3 (7)
	Tumor location		
	Central		8 (17)
	Upper inner		14 (30)
	Lower inner		2 (4)
	Upper outer		11 (24)
	Lower outer		1 (2 )
	Multicentral		10 (22)
	Immediate nipple reconstruction		6 (13)
	Mean operation time: min (range)	127 (61–270)	
	Mean specimen weight: g (range)		137 (36–300)
Pathology	Histology		
	Ductal		26 (57)
	Lobular		9 (20)
	Other		4 (9)
	DCIS		5 (11)
	Risk lesion		2 (4)
	Microscopic size (cm)		
	≤ 2		31 (67)
	> 2		15 (33)
	Multifocal tumors		26 (57)
	N+		11/39^a^ (28)
	ER+		35/39^a^ (90)
	HER2+		5/39^a^ (13)
	Ki-67		
	< 20		18/39^a^ (46)
	≥ 20		21/39^a^ (54)
	Lymph vascular invasion		7/39^a^ (18 )
	Mean smallest peripheral margin: mm (range)^b^		13.5 (3–25)
Adjuvant therapy	Radiotherapy	39 (98)	41(89)
	Medication		
	No medication	6 (15)	
	Endocrine treatment	15 (38)	
	Chemotherapy with/without endocrine treatment and anti-HER2 targeted therapy	19 (48)	
Neoadjuvant therapy		0	

*BMI* body mass index; *DCIS* ductal carcinoma *in situ;* *SLNB* sentinel lymph node biopsy; *N+* node-positive; *ER+* estrogen receptor-positive; *HER2* human epidermal growth factor receptor-2

^a^Among breasts with invasive cancers

^b^Excluding anterior and posterior margins, benign tumors’ margins

### Clinicopathologic Characteristics

The DLR technique was used for tumors located in any part of the breast. Most tumors (30%, 14/46) were located in the UIQ of the breast or in multiple quadrants (22%, 10/46). Axillary surgery was performed for 38 of 46 breasts, 6 of which required axillary clearance. Immediate nipple reconstruction was performed for six patients. The mean specimen weight was 137 g.

According to the final histopathologic report, 15 (33%) of the 46 tumors were larger than 2 cm, and 26 (58%) of the 46 tumors were multifocal. Three patients had triple-negative breast cancer.

### Surgical and Oncologic Outcomes

The surgical and oncologic outcomes are presented in Table 2. Negative margins were primarily achieved for 45 of the 46 breasts. Mastectomy was performed for one patient due to positive DCIS margins. Six patients had minor wound-healing problems. Three (50%) of six primarily reconstructed nipples had development of dermal necrosis and required bedside revision and sutures. Adjuvant treatment was not delayed for any of the patients with minor complications. Major complications occurred for three patients (6.5%, 3/46 breasts) who required reoperation, and adjuvant therapy was delayed for one of these patients.

**Table 2:** Breast morbidity and follow-up data

Complication	Clavien–Dindo classification adapted for breast cancer			Treatment *n* (%)
	Grade	Index breast (*n* = 46) *n* (%)	Contralateral breast (*n* = 26) *n* (%)	
No complication		34 (74)	26 (100)	
Minor	1	6 (13) Delayed healings		6 Local treatments, dressing
	2	0		
	3a	3 (6.5) Neo-nipple necroses		3 Bedside revision and suturing
Major	3b	2 (4.3) Hematomas 1 (2.2) Infected hematoma		2 Evacuations in the operating room and primary closure 1 Evacuation, debridement in the operating room, and secondary closure
	4	0		
	5	0		
Positive surgical margins in carcinoma or DCIS, breast				1 (2.1)
Delay of adjuvant treatment, patients				1 (2.5)
Mean time from surgery to adjuvant treatment: days (range)				35.5 (18–69)
Late corrections, patients^a^				2 (5)
Fat necrosis, breasts^b^				8/46 (17)
Mean follow-up: months (range)				44 (26–62)
Local recurrence, patients				1 (2.5)
Regional or distant recurrence, patients				0 (0)
Overall survival, patients				40/40 (100)

*DCIS* ductal carcinoma *in situ*

^a^One nipple reconstruction, one symmetry correction with nipple reconstruction

^b^Two palpable firmnesses < 3 cm without symptoms and five with symptoms, one palpable firmness > 3 cm with symptoms

Fat necrosis developed in eight breasts causing discomfort for seven patients. Late nipple reconstruction was performed for two patients, one of whom underwent additional reduction of the contralateral breast size. During a mean follow-up period of 44 months, one patient with triple-negative breast cancer experienced ipsilateral recurrence (at 22 months) and underwent mastectomy.

### Aesthetic Outcomes

**Fig. 3:**
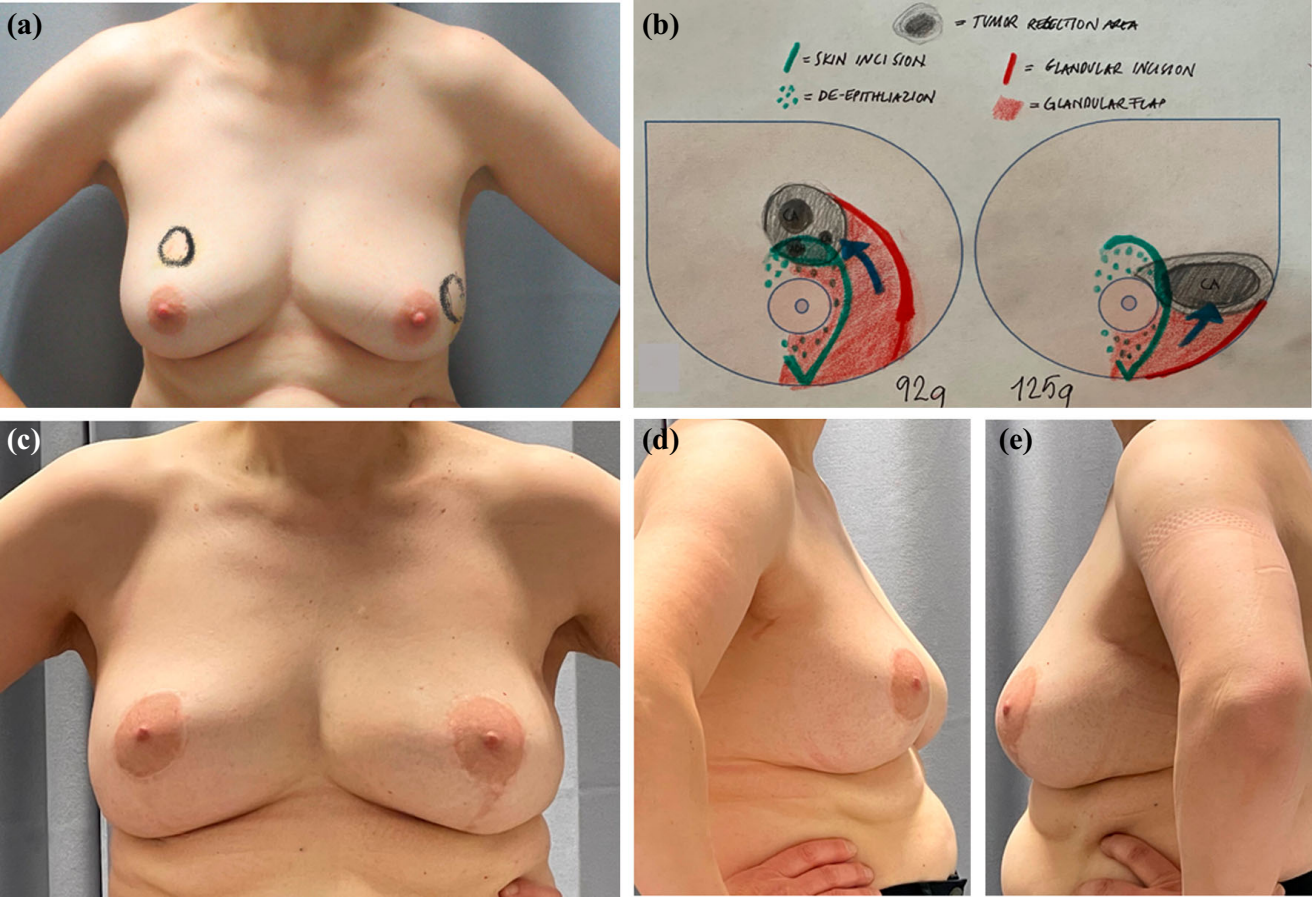
A 49-year-old woman with bilateral breast cancer (multifocal ductal carcinoma and atypical ductal hyperplasia in the right breast and a large 4.3 × 3.2 × 2.7-cm area of ductal carcinoma *in situ* associated with invasive ductal carcinoma in the left breast). The dual-layer rotation (DLR) technique was used on both breasts. **a** The tumor areas were ink-marked preoperatively on the skin. **b** Orientation map showing the tumors and planned resection areas in black, the glandular tissues to be manipulated in red, and the skin in green. The direction of glandular flap rotation is shown with blue arrows. **c**–**e** Postoperative photographs taken 2 years after surgery showing excellent aesthetic results according to BCCT.core software and subjective evaluation

Breast photographs for the aesthetic evaluation were obtained at least 1 year after radiotherapy. Three patients did not undergo surgical follow-up evaluation, and breast photographs were missing. The aesthetic results are reported in Table 3. The overall aesthetic results for both objective and subjective evaluation methods were between good and excellent regardless which part of the breast was managed by the DRL technique (Fig. 3). The objective results were worst for the patients without contralateral symmetrization.

**Table 3:** Objective and subjective aesthetic evaluation of all patients who underwent dual-layer rotation (DLR) and subjective aesthetic evaluation of breasts according to the tumor location and specimen weight

	Mean objective evaluations: BCCT.core (range)^a^	Mean subjective evaluations per patient: *n* (range)^a^
Patients (*n* = 37)^b^	3.2 (1–4)	3.4 (2–4)
Unilateral surgery (*n* = 7	2.0 (1–3)	2.9 (2–4)
Bilateral surgery (*n* = 30)	3.4 (2–4)	3.5 (2–4)

DLR technique, breasts (*n* = 41)	Tumor location	Mean subjective evaluations per breast: *n* (range)	Mean specimen weight: g (range)
Overall: *n* (%)		3.4 (2–4)	134 (36–300)
7 (17)	Central	3.4 (3–4)	89 (36–210)
13 (31)	Upper inner	3.2 (2–4)	128 (49–234)
2 (5)	Lower inner	3.5 (3–4)	162 (87–236)
8 (20)	Upper outer	3.5 (2–4)	104 (49–169)
1 (2)	Lower outer	4 (4)	80
10 (24)	Multicentral	3.4 (2–4)	195 (125–300)

*BCCT.core* Breast Cancer Conservation Treatment.cosmetic results

^a^Aesthetic evaluations were converted to a scale: 4 (excellent), 3 (good), 2 (fair), 1 (poor)

^b^Follow-up photographs were not available for three patients, two of whom underwent bilateral DLR

## Discussion

Large-volume resection of the medial areas of the breast, especially the UIQ, is challenging in terms of aesthetic outcomes for less ptotic, small, or moderately sized breasts, which usually require volume-replacement techniques if the breast is conserved. Therefore, the proposed DLR technique provides another possibility for treatment of upper and lower medial tissue defects using a full glandular rotating flap with a vertical tissue opening, avoiding other visible scars. Furthermore, with less rotation or smaller direction changes, the same technique can be used to fill defects in other parts of the breast. This proposed approach avoids skin resections except in the central areas close to the vertical incision.

Fitzal et al.^13^ previously described a surgical technique suitable for centromedial defects based on the vertical reduction mammoplasty and pedicle variability originally proposed by Hall-Findlay,^22^ which is similar to our DLR technique for central tumor defects. The proposed DLR technique can be adjusted for peripheral parts of the breast if more breast parenchyma is added to the vertical compensating flap from the inferior pole. Therefore, this DLR technique also can be used for lateral and inferior defects.

Compensation for superomedial defects can be accomplished by using different Wise pattern-based mammoplasties. The entire Wise pattern area, which normally is removed in reduction mammoplasties, can be used to fill tumor resection defects (e.g., by extending the inferior pedicle to the side in an upward direction), or it can be used as a secondary pedicle^8^ with or without the skin.

The Wise pattern mammoplasty techniques usually are reserved for large or ptotic breasts, and they are not very useful for breasts with a smaller volume or those with less excess skin. One third of the patients in this study had small breasts (A–B), which is not optimal for Wise pattern-opening. The scar burden also is relatively large when T-shape wound closure is applied in Wise pattern-based mammoplasties compared with vertical-based procedures, such as the DLR technique. Additionally, bat-wing and dermoglandular rotationplasty techniques can be used to compensate for small or medium-sized superomedial defects.^23^ Nevertheless, the disadvantage of these techniques includes rather long visible scars, which may extend to the decolte area, in which scars should be avoided if possible. Therefore, the vertical opening and tissue displacement in the DRL technique reduces the scar burden and offers more suitable aesthetic outcomes, even after large excisions.

Larger defects in the UIQ often require replacement material. Flaps that might reach the UIQ without microsurgical neovascularization can be raised from the upper abdominal or axillary regions as perforator flaps or as full or partial latissimus dorsi flaps with or without the skin. Alternatively, the tissues above the muscle can be raised as perforator flaps without the muscle, or the omentum can be used to fill tumor bed defects.^8^ These replacement methods require tissues adjacent to the breast and may increase the risk of morbidity and additional scars at the donor site but may in optimal situations keep the breast area scarless.^24^ For carefully selected patients, the DLR technique can offer a suitable alternative to volume-replacement techniques.

Re-excision after OBCS is technically challenging and has a negative impact on the patient’s care, psychological well-being, and cosmetic outcomes; increases healthcare costs; and might delay adjuvant therapy.^4,25–27^ Reoperations due to positive margins are relatively common after BCS (~ 20%), especially for patients with DCIS.^28,29^ The OBCS approaches allow resections with generous margins, thereby reducing the likelihood of positive margins (21% for conventional BCS and 12% for conventional OBCS).^30^ Nevertheless, multifocality and large lesion size are known risk factors for positive margins despite the use of OBCS techniques. In a large, prospective, international, multicenter study of therapeutic mammoplasty, O’Connell et al.^31^ reported incomplete excision of lesions in 132 (14.7%) of 899 breasts, of which 44.7% (59/132) had multifocal lesions, and the median size of the incompletely resected lesions was greater than that of the lesions with clear margins.

In the current study, the reoperation rate due to positive margins was low (2.1%), which presumably was multifactorial. All the preoperative studies were re-evaluated before surgery by specialized breast radiologists and multidisciplinary specialists. Every effort was made to evaluate the preoperative tumor extent and to transfer these findings to the surgical position by applying skin ink marks. Furthermore, all procedures were standardized, including tumor localization, macroscopic resection margins of 1 cm or larger, and en bloc resection to ensure negative anterior and posterior margins, all of which might contribute to the low frequency of positive margins. It was difficult to compare the reoperation rates between studies because of the wide heterogeneity in the study designs, inclusion criteria, and the methodology used. De La Cruz et al.^32^ reported a reoperation rate of 9% after OBCS when negative margins were defined as no tumor-on-ink, but the reoperation rate increased to 12.7% and 17.7% if the margins were 2 and 1 mm, respectively.

Complications also negatively affect the patients and their health care. More complex oncoplastic surgical procedures increase the risk of complications compared with simple lumpectomies but are associated with fewer complications than mastectomy.^4^ Nevertheless, to avoid poor cosmetic outcomes or mastectomy, complex OBCS is needed in aesthetically demanding areas or if high-volume resection is necessary. Additionally, secondary surgeries are considerably less frequent after OBCS than after skin-sparing mastectomy combined with primary reconstruction.

The frequency of complications was relatively high in our study (26%). The vast majority of the complications were minor (19.5%), although the complication rate was higher than the 14.3% reported in the systematic review by De la Cruz et al.^32^ In our study we used the standardized CDC classification, which may be sensitive to detecting minor complications.

Similar to our results, the frequency of complications in prior studies ranged from 25.9 to 30.8%.^16,33,34^ The frequency of major complications (6.5%) in our study was comparable with the frequencies of 0% and 8.6% in prior studies.^35,36^ Nevertheless, the mean time from surgery to the start of adjuvant treatment in our study was 36 days, consistent with two prior studies, in which the mean values were respectively 30 and 47 days.^37,38^ The minor healing problems recorded in the current study did not adversely affect the start of adjuvant therapy, which was delayed for only one patient with major complications.

Extensive mobilization, especially in fatty glandular tissue, carries a risk of fat necrosis as a late complication of OBCS.^3,39^ Fat necrosis is reportedly found in 4% to 26% of OBCS patients.^40,41^ Fat necrosis can cause discomfort and pain for patients, possibly resulting in skin color changes and breast distortion. Palpable or radiologically detected fat necrosis can resemble cancer recurrence, causing patient anxiety and increasing the biopsy rate, which is known to be higher in OBCS than in standard breast conserving surgery (S-BCS).^4^ In our study, fat necrosis was relatively common after the DLR technique (17%), mainly classified as minor with symptoms,^17^ showing results comparable with those reported by Dolan et al.^42^ Avoiding DLR for breasts with scattered fibroglandular tissues or fatty breasts may minimize the risk of fat necrosis, as was also reported by Zaha et al.^39^ for their level 1 OBCS technique.

Although self-perceived body image seems to be a more important determinant of quality of life than the cosmetic outcome after breast cancer surgery,^43^ large resection (weight >75 g), scar visibility, and resection of the inner half of the breast or behind the NAC are associated with less satisfactory aesthetic outcomes.^44–46^ The DLR technique used in this study provided good aesthetic outcomes despite the tumor location in the upper inner, central, or multicentral positions; the large specimen sizes; and the small breast sizes in one third of the patients. The patients who declined the offer for procedures to be symmetrized tended to have worse aesthetic outcomes.

Our study had some major limitations. It was a single-center retrospective study, and no patient-reported quality-of-life outcomes were available for evaluation or comparison with the image-based aesthetic results. The DLR technique was not used for the patients who needed skin excision outside the central area, which might have compromised the skin envelope vascularity or the final aesthetic results. Further research is needed to examine the possible role of DLR in skin resection if oncologic skin removal is indicated. Despite these limitations, we have reported a novel OBCS approach that might improve the aesthetic outcomes or avoid mastectomies for selected patients with large tumors, challenging lesion locations, or both.

In conclusion, the DLR technique is a novel one-step procedure that can be used to treat selected breast cancer patients with large lesions or challenging defects not only in the medial parts of the breast, but also in central and lateral locations. The procedure also can achieve a natural appearance for smaller breasts.
